# The WHO construct of health-related functioning (HrF) and its implications for health policy

**DOI:** 10.1186/1471-2458-11-S4-S9

**Published:** 2011-05-31

**Authors:** Luis Salvador-Carulla, Carlos Garcia-Gutierrez

**Affiliations:** 1 Research Unit, Fundació Villablanca. Grup pere Mata, Reus, Spain; 2 Mental Health Unit, Hospital de Puerto Real, University of Cadiz, Cadiz, Spain

## Abstract

**Background:**

The International Classification of Functioning (ICF) has acquired a central role in the WHO Family of International Classifications and it has been extensively adopted as the reference framework for health-related functioning (HrF). This review aims to provide a description of the ICF/HrF to contextualise ICF/HrF in relation to other approaches to health functioning and to describe its application in policy and legislation with a special focus on Spain.

**Methods:**

Narrative review based on the scientific literature and prior expert knowledge.

**Results:**

ICF is both a coding system and a conceptual framework of HrF, which is framed as a unidimensional, bipolar and asymmetric construct with a negative pole (disability) and a positive pole (good functioning) with higher complexity. Other models of HrF include health promotion, quality of life and activities of daily living (ADL). The curtailed taxonomy of ICF and its unclear distinction from other approaches have had significant implications for research, policy and legislation, as illustrated by the case of the legislation and services for functional dependency in Spain and other examples.

**Conclusions:**

The ICF model of functioning is more comprehensive and usable than previous alternatives, but a full taxonomy of the HrF construct is needed to avoid further confusions in this field. This should also comprise harmonisation with other classifications of the WHO Family of International Classifications and other models of health functioning.

## Background

Health-related functioning (HrF) plays an increasing role in medicine. Assessment of functioning and disability is critical to understand the relationship between the individual and the disease [1], functional status indicators provide a robust predictor of health events [2], and the predictive power of disability exceeds that of clinical diagnosis in many chronic conditions. However, “disability” is still an elusive concept in medicine [3], and there is also a complex relationship between the construct of functioning/disability and the concepts of autonomy and dependency which requires rigorous taxonomical analysis [4].

Since its release in 2001, the International Classification of Functioning (ICF) [5] has progressively acquired a central role in the WHO Family of International Classifications (WHO-FIC), as the logical connexion between the ICD subgroup (International Classification of Diseases) and the classifications of contextual factors, such as the International Classification of Health Interventions (ICHI)[6], or health services at the System of Health Accounts (SHA 2.0) [7].

ICF is indeed a proto-taxonomy of the components (determinants, factors and consequences) of health conditions. A decade after its publication, ICF has produced an intense conceptual debate. It has also generated a comprehensive battery of instruments which clearly prevails over previous efforts in the assessment of functioning and disability.

ICF has been adopted by many countries and international organisations as the reference framework for classification, data register, health and social policy and legislation on disabilities and related areas. However, the ICF framework has been loosely used in policy and legislation, and the lack of a rigorous application of the ICF framework and the ICF classification has had a great impact on the assessment and disability policy both at regional and national level.

The aims of this review are 1) to provide a narrative review of the ICF concept of HrF; 2) to contextualise this concept in relation to other approaches to functioning currently used in Medicine; and 3) to describe several cases that illustrate its application in policy and legislation with a special focus on Spain.

## Methods

A narrative review has been carried out based on purposively selected scientific literature and official reports, and on the author’s prior expert knowledge in the field. This knowledge is based on his experience as member of the Spanish group for the development of ICF, the development of the Spanish system for assessment of services for disabilities and dependency (DESDE) [8] and his role as member of the Advisory Council on Dependency of Catalonia (Spain), as well as previous research in the field.

First, the ICF is described in its double role as a coding system and as a conceptual framework of HrF. Secondly, the relation of ICF/HrF with other models of health functioning (Health promotion, Quality of Life and Activities of Daily Living) is examined, and several cases of its use in legislation and policy are presented.

### The ICF concept of Health-related Functioning (HrF)

Although ICF is accepted internationally as a groundbreaking and comprehensive system, several criticisms have been posed. Critics refer largely to: 1) overall usability of the system; and 2) taxonomy problems mainly related to the distinction between activity and participation, capacity and performance, and its granularity and need for additional qualifiers [4]. A relevant unforeseen aspect of ICF is that it is both a classification/coding of HrF and at the same time the conceptual framework of the WHO HrF construct. These two roles of ICF are frequently confused and this confusion has significant implications for research, policy and legislation.

### ICF as a classification and coding system

ICF is a revision of the previous 1980 WHO International Classification of Impairment, Disability and Handicap (ICIDH), with a core coding system for body structures and functions (and their related impairments), activities (and their related limitations formerly called “disabilities”) and participation (and their related restrictions previously called “handicaps”). It also provides a complementary and less developed classification of the environmental “components” of functioning [5].

Being a major advance over ICIDH, it is important to note that ICF has usability and taxonomy problems. Although a full appendix on “Taxonomic and Terminological Issues” was included in the ICF book, there are problems in its hierarchical structure and granularity that are mainly related to a truncated taxonomy and lack of a formal ontology approach when the classification was made [4], and probably to the consensus adopted between experts and stakeholders to sort out different perspectives on its biopsychosocial background before ICF was finally endorsed by the Fifty-fourth World Health Assembly in 2001.

With regard to its usability, ICF “does not classify people, but describes the situation of each person within an array of health or health-related domains” (ICF, page 8). As it may be easily understood, while this could be useful for individual care planning, it is a major challenge for the usability of ICF as an administrative and policy tool to guide accessibility to services and benefits, for its use in surveys and national databases, and for other purposes related to health and social care planning. Even so, a series of initiatives have been put forward to improve the usability of ICF addressed to facilitate the assessment of people.

The Mini-ICF-P is a short observer rating instrument for the assessment of disabilities, especially with regard to occupational functioning [9]. An ICF check-list and ICF Core sets are also available for an increasing number of diseases. Core sets are subgroups of ICF items selected to capture those aspects of functioning that are most likely to be affected by specific disorders. Within a given disorder, both Brief and Comprehensive Core Sets can be established to serve specific purposes. There are also efforts to develop a generic ICF core set. Its proponents argue that although the specific core sets are useful for describing particular conditions, the generic set will be valuable to compare across health conditions, serving as a common language based on the principle of “etiologic neutrality” [10]. The new and expanded version of the WHO Disability Assessment Schedule (WHODAS-II) was developed to assess disability in medical conditions and it is considered as part of the ICF battery [11].

The development of a formal ontology of the ICF classification has been recently started by the IFC Ontology ICT group [12]. A new version has been developed for children and adolescents (ICF-CY) [13] which incorporates relevant changes. A standard definition of “impairment” is provided, and several dimensions such as “mental functions” have been expanded.

Other major challenges of ICF as a taxonomy remain unsolved. As an example, there is an overlap between the concept of “impairment” in ICF and “symptoms and signs” in ICD (ICF, page 4):

“It is also important to recognize the overlap between ICD-10 and ICF. Both classifications begin with the body systems. Impairments refer to body structures and functions, which are usually parts of the “disease process” and are therefore also used in the ICD-10. Nevertheless, ICD-10 uses impairments (as signs and symptoms) as parts of a constellation that forms a “disease”, or sometimes as reasons for contact with health services, whereas the ICF system uses impairments as problems of body functions and structures associated with health conditions”.

Another unsolved conflict is the supraordinal classification of the domains activity and participation. ICF uses the same “d” code for both domains, which can be used either together or separate. This choice produces a critical taxonomic conflict in any classification system, and it may reflect tension between experts in the field and family and user organizations during the development process of the ICF. As a matter of fact, it is the only classification of the WHO-FIC that allows four completely different uses of its core coding system (ICF, page 16):

“It is difficult to distinguish between "Activities" and "Participation" on the basis of the domains in the Activities and Participation component. Similarly, differentiating between “individual” and “societal” perspectives on the basis of domains has not been possible given international variation and differences in the approaches of professionals and theoretical frameworks. Therefore, ICF provides a single list that can be used, if users so wish, to differentiate activities and participation in their own operational ways. This is further explained in Annex 3. There are four possible ways of doing so:

(a) to designate some domains as activities and others as participation, not allowing any overlap;

(b) same as (a) above, but allowing partial overlap;

(c) to designate all detailed domains as activities and the broad category headings as participation;

(d) to use all domains as both activities and participation”.

The ICF qualifiers have received great attention in the literature. The use of the capacity and performance qualifiers have been extensively described in the recent years [14,15]. Less attention has been paid to barriers and facilitators (and incentives). Addressing facilitators and barriers may help experts to guide priorities for interventions. Linking interventions to aspects of participation valued by the patient/client seems to make a very real difference in promoting engagement in processes like goals and goal setting [16]. Additional qualifiers such as opportunity, control and will (volition and self-efficacy) have also been suggested [17-19] , although these may be regarded as part of the personal factors that have yet to be described and coded (e.g. volition and self-efficacy). However, the interaction of these factors may be extremely complex. For example, discriminatory practices are barriers that prevent the performance regardless of capacity. This external/environmental factor has a clear impact on many personal factors including volition.

On the other hand, the rehabilitation research (which seeks to understand how to change specific aspects of function), and enablement/disablement research (which seeks to understand how changes in one part of the ICF framework affect functioning elsewhere), and the “proximal and distal” consequences of impairment, as suggested in the brain injury field, may require a careful appraisal in the next future [20].

### ICF as a model of Health-related Functioning (HrF)

**Figure 1:**
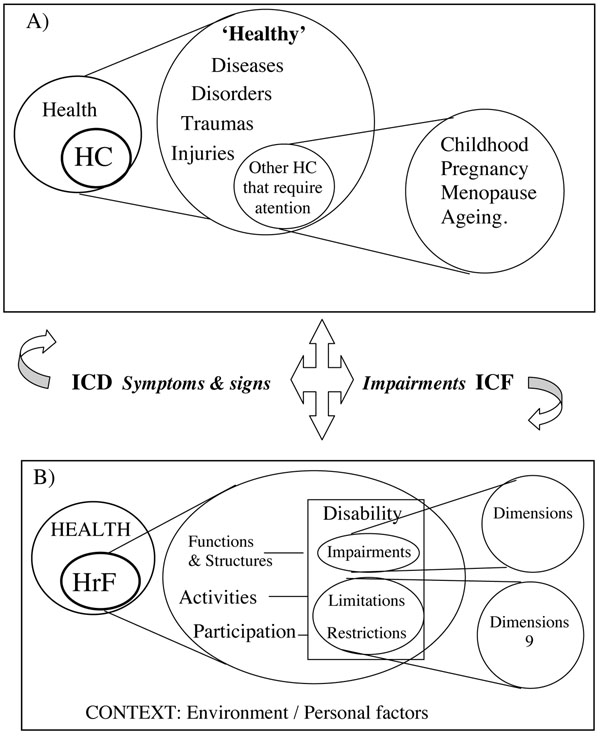
**Hierarchical map of health conditions and health-related functioning including several perspectives from WHO: Health promotion, Health-related Quality of Life, and Health-related functioning based on ICF** A) health conditions and B) health-related functioning.

Functioning and disability (D&F) are two related domains of the construct “health-related functioning”. We recently made a comprehensive review of this construct and a series of related concepts in ICF and other WHO documents [4] . D&F may be initially regarded as a unidimensional bipolar construct with a positive pole (good functioning) and a negative pole (disability) in ICF. However, there is a clear asymmetry between the two poles, as positive functioning involves many more alternatives than negative functioning. On the other hand, the analysis of the hierarchical structure and the conceptual relationship between the terms ‘functioning’ and ‘disability’ in the WHO family of classifications and related documents may indicate that ‘disability’ is actually a subcategory of ‘functioning’, as ‘disease’ is a subcategory of ‘health condition’ (Figure 1). The current definition of ‘functioning’ in ICF should incorporate the wording ‘positive functioning’ and “health-related” functioning as the global term, as health-related quality of life is regarded as a subcategory of well-being.

**Figure 2:**
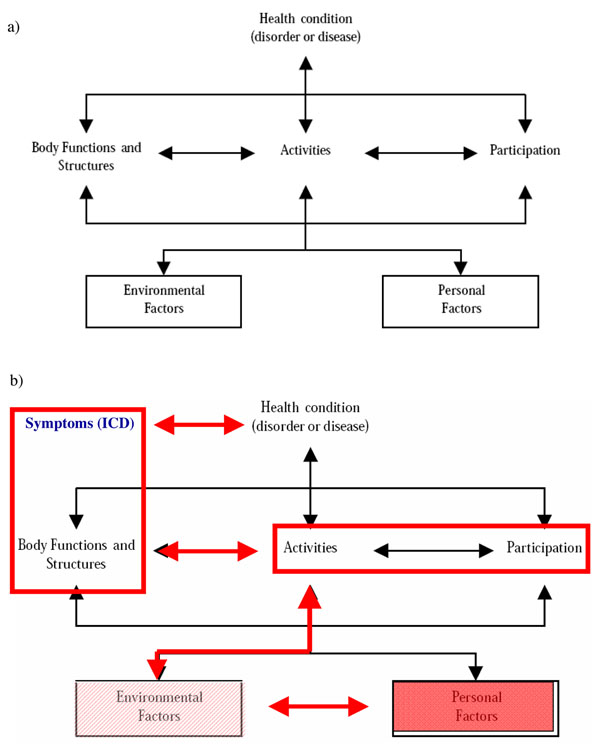
**Differences between ICF as a model of functioning and as a coding system**. a) Theoretical interaction of the components of the “process of functioning” model of ICF; b) actual relations among the different components in the ICF classification system (environmental factors are partially described, whilst personal factors are not coded by the ICF).

In this review we focus on the taxonomical aspects of the ICF/HrF and its boundaries with other related models in the health sector. It is important to note that the biopsychosocial approach which is mentioned in the ICF to capture the different perspectives on functioning (medical and social) is not formally defined, and it has been surpassed by the holistic/integrative model [21]. In any case, the “functioning” assessed by ICF is health-related functioning and this should be made clear to avoid misinterpretation, even though the ICF diagram of the process of functioning clearly states this hierarchical relation. However, it is also important to note that the ICF model of the “process of functioning” is only partially described in the ICF book, although its main components and the existing relations among them are clearly established (Figure 2a).

This ambiguous approach to the underlying model is strengthened by the use of the term “model” with different meanings in different sections of the ICF and by the statement made at page 18:

“As a classification, ICF does not model the “process” of functioning and disability. It can be used, however, to describe the process by providing the means to map the different constructs and domains”.

The “process of functioning” diagram does not actually correspond with the ICF classification system. The link between “body functions and structures” and “health conditions” is stronger than the link between activities and participation, as impairments and symptoms are quasi-equivalent terms in ICF/ICD. This link has been referred to as the biomedical model approach by Stucki and colleagues [22] in contrast with the integrative/biopsychosocial approach promoted by the ICF model and its related diagram.

However, the hierarchical order in this diagram and the statement about the relationship between symptoms and impairments in ICF really identifies a closer link between health conditions and impairments than with activities and participation. Furthermore, a whole domain of the process of functioning model is not coded in the ICF (personal contextual factors) and the environmental domain is only partially described. For example, the coding of health services -e5800-, does not match the related WHO classification of health functions (SHA 2.0) [7], whilst interventions classified in ICHI are not even mentioned as environmental factors in ICF. A tentative representation of the actual relations among the different domains of the “process of functioning” model is provided in Figure 2b.

### ICF-HrF and other models of functioning

Before the inception of ICF and its HrF model, three other models of functioning were broadly used in medicine: the Health promotion model, the Quality of Life (QoL) model and the Activities of Daily Living (ADL) model. From a conceptual point of view HrF has surpassed them, as it provides a framework that directly connects functioning to the underlying health condition (ICD) as well as to the services (SHA 2.0) [7] and interventions (ICHI) [6] needed, among other contextual factors whose classification is under development. It also provides a positive approach on activities and participation which, paradoxically, was taken into account in the QoL model and which was not connected to health conditions in the Health promotion model.

### Health promotion model and functioning

The WHO approaches to health promotion and ageing offer complementary concepts of great importance for the development of a more complete taxonomy of HrF. The Ottawa Charter for Health Promotion [23], the related Jakarta Declaration for Health Promotion in 1997 and its glossary [24], and several WHO documents on Ageing and Active Ageing [25,26] have clarified the existing relations between health and disease and have provided a life-span approach and a positive health perspective. These are critical to a better understanding of the relationship between health and functioning as shown in Figure 1 and illustrated by the conceptualisation of independence/dependence and positive health-related functioning and the complex relation between environmental and personal factors described in the social capital model of positive health functioning.

The WHO glossary of terms for community health care and services for older persons [27] defines “independence” as the “ability to perform an activity with no or little help from others, including having control over any assistance required rather than the physical capacity to do everything oneself”. This concept may be applied to produce a WHO definition of functional dependency, which is needed to encompass current developments in legislation and service provision for “dependency” discussed in the next section with the ICF model.

The World Health Organisation has set up a framework for the conceptualisation of health promotion and public health [28,29]. This report emphasises positive health and social capital as well as a cultural sensitive approach which takes into account the resources available across countries. Mental health promotion aims to impact on determinants of health so as to increase positive health, to reduce inequalities, to build social capital, to create health gain and to narrow the gap in health expectancy between countries and groups [29]. The health promotion model is also part of the WHO conceptual system and, although a formal connection with ICF has not been established, it played a major role in incorporating the positive approach to health and it provides a sound background for the classification of health-related habits and lifestyle [30], which are mentioned in ICF as key components of the personal contextual factors in the “process of functioning” diagram (Figure 1).

The definition of promotion used in Europe has a slightly broader scope as it specifically mentions positive functioning as a key component. It includes activities that aim to protect and support “emotional and social well being and create the conditions that enable optimal functioning of individuals, families, communities, and societies” [31].

Social capital is a key domain of health promotion. Recently this concept has been revised to incorporate a more holistic approach. The “Mental capital” approach has significant implications for the development of promotion/prevention strategies worldwide [32] and it is a relevant model for framing positive functioning together with the integrative approach to healthcare [21]. Mental capital refers to “the totality of an individual’s cognitive and emotional resources, including their cognitive capability, flexibility and efficiency of learning, emotional intelligence and resilience in the face of stress. The extent of an individual’s resources reflects his or her basic endowment (e.g. genes and early biological programming), motivation and experiences (e.g. education) which take place throughout the life course.” The Foresight Mental Capital and Wellbeing Project (a UK Government project in the Government Office for Science) was launched to promote mental capital initiatives in the population and in the health care system [33]. A series of simple recommendations were related to this program and linked to the positive aspects of wellness. They were summarised as “steps to happiness” for a media campaign (Henderson M, The Times, October 22, 2008). The 5 steps are as follows: 1) connect (developing relationships with family, friends, colleagues and neighbours will enrich your life and bring you support); 2) be active (sports, hobbies such as gardening or dancing, or just a daily stroll will make you feel good and maintain mobility and fitness); 3) be curious (noting the beauty of everyday moments as well as the unusual and reflecting on them helps you to appreciate what matters to you); 4) learn (fixing a bike, learning an instrument, cooking – the challenge and satisfaction brings fun and confidence); and 5) give (helping friends and strangers links your happiness to a wider community and is very rewarding). As it may be figured out, the social-mental capital provides a framework for understanding the existing relations between activities (e.g. learning), environmental factors (social network, healthy communities) and the positive personal factors related to them, and this approach may be incorporated to relational models which provide a dynamic approach to ICF (e.g. friction model) [15].

### QoL model and functioning

The content validity of the ICF model in relation to the QoL construct has been previously analysed [34]. QoL is defined by WHO as “individuals' perceptions of their position in life in the context of the culture and value systems in which they live and in relation to their goals, expectations, standards and concerns” [35]. Apparently the QoL concept shows little overlap with the ICF, however all health-related QoL instruments include self-perceived negative functioning as a key component of the QoL construct, together with the assessment of overall ratings on health and specific symptoms. This approach was also followed by the WHOQOL [36], and by its latest version, the WHOQOL-SRPB, which also includes personal factors such as spirituality, religiousness and personal beliefs [35]. Although HQoL was a great improvement of health assessment during the last third of the twentieth century, the development of a full HrF construct has surmounted the QoL approach, which should be decomposed in its different parts and assessed and analysed separately. The “deconstruction” of the QoL approach is illustrated by the use of subscales of SF-36 as indicators of functional impairment and performance [14,37], and by its use for developing mental health indicators in the European Union [38]. In any case, knowledge transfer within WHO is urgently needed as regards the relation between WHOQOL and ICF/HrF, as well as between related research groups [39].

### ADL model and functioning

The ADL model was originated in the US right after World War II to measure functioning in cancer patients and in physical rehabilitation [40]. In the 1960s, Katz [41] and Lawton and Brody [42] distinguished two major groups of ADL: “basic” activities related to self-care, such as eating and grooming (BADL); and “instrumental” activities, such as cooking and handling money (IADL). This approach was used to develop the Katz ADL index [43] and the Barthel index [44], which is still a standard rating scale to measure disability in geriatrics and other medical disciplines as well as the standard comparator to assess the psychometrics of related instruments [45]. In spite of its inconsistencies, the distinction between BADL and IADL is still deeply grounded in the medical assessment of disability [46]. While in ICF disability is linked to global functioning, in the ADL model it is linked to impairment in a reduced set of ADL. In physical conditions, ADL and ICF models may produce convergent results, whilst significant differences appear in mental disorders. In severe mental illness, high social support may be needed even when there is hardly any impairment in “basic” ADL.

### Applications of the HrF to health policy

The concept of ‘functional dependency’ derived from the ADL model in the early 1990s has inspired legislation on care for older persons in many Western countries [4]. It was later extended to other groups with severe disabilities and it has provided an international framework for evaluation and care of frail population across the lifespan in countries such as the US [46], Japan [47], Mexico [48], or the European Union [49].

In 1998, the European Council made a recommendation to EU member states to develop care for dependent which was based on the ADL approach. The Council of Europe defined ‘dependency’ as the condition related to the loss of autonomy and the need of support from a third person due to impairment of activities of daily living, especially self-care. Later on, and as the European countries adopted ICF, the dependency approach was linked to the ICF model. It was implicitly assumed that ADLs and related assessment instruments fitted the ICF model without even taking into account that words such as “basic and instrumental ADLs” and “dependency” are not even mentioned in the ICF. This may explain the fact that, in many European countries, the legislation on functional dependency is theoretically based on ICF while it uses the ADL approach for the assessment and for the development of eligibility criteria to different levels of care provision [4].

Unfortunately, current approaches based on QoL or ADLs have failed to provide a workable case-mix related to functional dependency in countries such as Spain, France or Germany; particularly, in severe mental illness [50]. This is partly due to the complexity of the concept of functional dependency, which has been described as a meta-construct involving the constructs functioning/disability, personal support and care needs [4]; to the different indicators related to these constructs (clinical status, functional impairment, quality of life, objective and subjective burden, service use, care needs, etc); and to the additional complexity of mental disorders, where disabilities are not just related to activities of daily living but to other aspects of general functioning such as social isolation, low medication adherence and behavioural problems requiring intensive surveillance by carers [51]. The ADL/dependency approach has also failed to provide international comparability at least in Europe [49].

In Spain, the Law for the promotion of personal autonomy and care for persons with dependency (LPAD 39/2006, 14th December) was approved by the government in 2006 and enacted from 2007. This Law was theoretically based on the ICF model. Regional agencies of Dependency were progressively implemented in the Autonomous Communities (regions) of Spain thereafter, and a national instrument was designed for assessing eligibility to related benefits and access to special services. A series of documents prepared by some regional agencies (e.g. PRODEP in Catalonia), the National Disability Council (known by its Spanish acronym, CERMI) and several NGOs warned IMSERSO (the national agency responsible for the dependency programme) that ICF was not being followed by the dependency system, and that the assessment instrument was based on the Barthel Index derived from the ADLs model and that there were alternatives based on ICF. Regardless of these warnings the system was implemented in 2007. It was designed as a social support system with a secondary role in the health sector and an exclusion of the well experienced centres for the official assessment of disabilities which was mostly based on the 1980s ICIDH and which were at that time adapting the ICF [51].

In 2008, the Spanish Congress designed an Expert Group to make a surveillance of the enactment of the LPAD. The report released by the Group in September 2009 [52] and other external data pointed out the failures of the assessment process, with a great impact on the efficiency and feasibility of the dependency care system in Spain. However, the underlying conceptual problem was not mentioned in the report. There is now a high variability in the eligibility and reporting of dependency across the 17 regions or “Autonomous Communities” in Spain [53].

These conceptual aspects, the need for a major involvement of the health sector, and the actual adaptation to the ICF model have been suggested by other authors [51,54,55].

Curiously enough, the Spanish dependency agency IMSERSO states that the assessment procedure is already adapted to the ICF model regardless of the reports and the institutional requests made by several Spanish organisations. This may reflect problems in the ambiguous formulation of the ICF model mentioned above, and the need to properly define its boundaries with other approaches such as ADL.

The confusion of ICF with other approaches to health functioning may be found in other international classifications. As an example, the May 2010 draft of the System of Health Accounts (SHA 2.0) [7] produced by OECD with the collaboration of WHO National Health Account System, initially incorporated the BADL/IADL subtyping for the description of nursing care.

Another interesting case is the classification of intellectual disability developed by the American Association of Intellectual and Developmental Disabilities (AAIDD), which is the main specific classification in this field and has been broadly accepted in Spain and other EU countries. It is theoretically based on the ICF model whilst it is mainly grounded on a previous, long-standing conceptual frame based on the “positive appraisal” of skills/adaptive behaviour that is not considered as such in ICF. In 1992, the classification system developed by the AAIDD (formerly called AAMR for ‘Mental Retardation’) released a classification system which provided a specific dimension to assess the “adaptive behaviour” in Intellectual Disabilities [Dimension II: Adaptive behaviour, which includes three domains (conceptual, social and practical skills), 16 types, and 26 skills or adaptive behaviours]. This approach has been extended in the current 11th edition of the AAIDD classification following the same paradigm [56]. These are personal contextual factors not currently described in the ICF. The AAIDD classification also incorporates a system to evaluate the level or intensity of support needs and the planning process to implement these supports, which may be linked to the “facilitators” qualifier in the ICF.

## Conclusions

ICF has been one of the major advances of integrative health assessment and classification at the beginning of the twenty-first century. The richness and possibilities of ICF are now clearer than when it was conceived. Its separate role as a paradigm of health-related functioning and as a classification and coding system of the functioning “components” of health is know better understood. However, ten years after its publication, a critical review is urgently needed as well as a thorough revision of its taxonomy and its formal ontology.

In this paper, we have reviewed a series of key challenges faced by the ICF model which deserve further attention both by WHO and the different ICF working groups. This challenges were partly ignored in the past not to impede a global consensus on the fundamentals of the ICF/HrF paradigm. However, this position has led to major problems in the application of ICF to health and social policy and legislation in different countries, as well as to problems in adopting the ICF/HrF conceptual framework in other specific classification systems.

Bridging and knowledge transfer of the different approaches within WHO to HrF (e.g. WHOQOL, Ageing, Health promotion, and other classification systems) is urgently needed, as well as the development of a coherent WHO conceptual system, and further efforts to better clarify and harmonise ICF/HrF to international legislation on functional dependency among other initiatives.

## Competing interests

The authors declare that they have no competing interests.
